# Association Between Human Papillomavirus Vaccination and Primary Ovarian Insufficiency in a Nationwide Cohort

**DOI:** 10.1001/jamanetworkopen.2021.20391

**Published:** 2021-08-26

**Authors:** Anders Hviid, Emilia Myrup Thiesson

**Affiliations:** 1 Department of Epidemiology Research, Statens Serum Institut, Copenhagen, Denmark; 2 Pharmacovigilance Research Center, Department of Drug Design and Pharmacology, University of Copenhagen, Copenhagen, Denmark

## Abstract

**Importance:**

Anecdotal case reports have suggested an association between human papillomavirus (HPV) vaccination and primary ovarian insufficiency, but observational studies of HPV and primary ovarian insufficiency are rare, and their findings do not support an association. However, available studies have been limited by statistical power, and concerns about infertility after vaccination are associated with lower levels of uptake of the cancer-preventing vaccine in many countries.

**Objective:**

To evaluate the risk of primary ovarian insufficiency after quadrivalent human papillomavirus (4HPV) vaccination.

**Design, Setting, and Participants:**

This retrospective cohort study with follow-up from 2007 to 2016 used nationwide data for 996 300 Danish-born girls and women aged 11 to 34 years. Cox proportional hazards regression was used to estimate hazard ratios (HRs) of primary ovarian insufficiency diagnoses by 4HPV vaccination status with adjustment for age, calendar period, and a propensity score summarizing health care use. Data were analyzed from October 2020 to January 2021.

**Exposures:**

Receiving 4HPV vaccination compared with receiving no vaccination.

**Main Outcomes and Measures:**

The main outcome was hospital contacts for primary ovarian insufficiency, and the main outcome measures were HRs comparing rates of primary ovarian insufficiency among vaccinated and unvaccinated individuals.

**Results:**

During 6 781 166 person-years of follow-up among 996 300 girls and women aged 11 to 34 years (505 829 vaccinated individuals [50.8%] and 490 471 unvaccinated individuals [49.2%]), 144 individuals were diagnosed with primary ovarian insufficiency, including 54 individuals diagnosed after 4HPV vaccination. The median (interquartile range) age of primary ovarian insufficiency diagnosis was 26.94 (12.68) years. The adjusted HR of primary ovarian insufficiency comparing 4HPV vaccination to no vaccination was 0.96 (95% CI, 0.55-1.68).

**Conclusions and Relevance:**

This study found no association between HPV vaccination and primary ovarian insufficiency. However, given the rarity of the outcome in this study, the presence of a clinically relevant increase in rate of diagnosis cannot be excluded.

## Key Points

**Question:**

Is human papillomavirus vaccination associated with primary ovarian insufficiency among Danish girls and women?

**Findings:**

In this cohort study of 996 300 girls and women, vaccination was not associated with primary ovarian insufficiency.

**Meaning:**

This finding suggests that human papillomavirus vaccination is unlikely to be associated with moderate to large increases in the risk of primary ovarian insufficiency.

## Teaser

This cohort study investigates the risk of primary ovarian insufficiency after human papillomavirus vaccination among Danish girls and women.

## Introduction

In many countries, the acceptance of routine human papillomavirus (HPV) vaccination has been hampered by spurious safety concerns.^1,2^ One of the key concerns has been the claim that HPV vaccination is associated with infertility through primary ovarian insufficiency. This claim surfaced in 2012 from an anecdotal case study of a 16-year-old girl who developed primary ovarian insufficiency after receiving an HPV vaccination.^3^ A number of other case reports followed, comprising 5 additional cases.^3,4^ Unfortunately, case reports like these sometimes gain an amount of attention in the press and on social media that does not always correspond to their scientific significance. Suggested biological mechanisms for an association between HPV vaccination and primary ovarian insufficiency have been the induction of autoimmunity to ovarian cells by immune stimulation and toxic effects associated with vaccine ingredients.^5,6^ Primary ovarian insufficiency is a rare outcome, and an evaluation of its association with HPV vaccination necessitates large observational data sources. Currently, to our knowledge, 1 observational study has evaluated the association. Naleway and colleagues^7^ evaluated the association between adolescent vaccinations, including HPV vaccination, and primary ovarian insufficiency in a cohort of 199 078 girls and women from a US health maintenance organization. A total of 46 individuals were included in the study, and 1 diagnosis occurred after HPV vaccination, yielding an adjusted hazard ratio (HR) of 0.30 (95% CI, 0.07-1.36). Passive surveillance suggests that primary ovarian insufficiency is rarely reported. Arana and colleagues^8^ found a rate of 0.28 reports of physician-diagnosed primary ovarian insufficiency per 1 million distributed doses of quadrivalent HPV (4HPV) in the US Vaccine Adverse Event Reporting System. We conducted a nationwide cohort study investigating the association between HPV vaccination and primary ovarian insufficiency in Danish girls and women aged 11 to 34 years during 2007 to 2016 with historically prospective follow-up.

## Methods

### Study Cohort

We took advantage of the unique opportunities in Denmark for register-based research to construct a nationwide cohort of all Danish-born girls and women aged 11 to 34 years during 2007 to 2016. The construction of a nationwide cohort was made possible by the existence of the Danish Civil Registration System.^9^ This register contains information on date of birth, vital status, civil status, and place of residence for every individual residing in Denmark indexed by a unique identifier. This unique personal identifier is used in all other national registers, including registers containing extensive health care information. Using the identifier, we were able to link information on HPV vaccination status, potential diagnoses of primary ovarian insufficiency, and health care use with each individual in the study cohort. The study was approved by the Danish Data Protection Agency. Neither ethical approval nor informed consent is required for register-based research in Denmark. This study followed the Strengthening the Reporting of Observational Studies in Epidemiology (STROBE) reporting guideline for cohort studies.

### Vaccination

We obtained information on dates of HPV vaccination in the study cohort from the Danish vaccination register.^10^ The 4HPV vaccine (Gardasil, Merck Sharp and Dohme) was introduced in the Danish childhood immunization schedule in January 2009. Initially, girls aged 12 years were offered the vaccine in a 3-dose schedule. Girls aged 13 to 15 years were initially offered catch-up vaccination in October 2008. The offer of catch-up vaccination was later extended to women aged 20 to 27 years in August 2012. The bivalent vaccine (Cervarix, GlaxoSmithKline) replaced the quadrivalent vaccine in February 2016. The Danish vaccination register comprises HPV vaccinations administered within the framework of the free national childhood immunization program and HPV vaccinations privately purchased and administered outside of the program for older individuals not eligible for the national program. We excluded individuals vaccinated before study start.

### Primary Ovarian Insufficiency

Diagnoses of primary ovarian insufficiency among individuals in the study were ascertained from the Danish National Patient Registry.^11^ This register contains individual-level information on hospital contacts with assigned diagnoses coded using the *International Statistical Classification of Diseases and Related Health Problems, Tenth Revision* (*ICD-10*). We used the following diagnoses codes: E28.3 (ie, primary ovarian hypofunction) and E28.3A (ie, premature menopause). In the Danish setting, general practitioners act as gatekeepers to specialist care and the hospital. We included all types of hospital contacts, and primary and secondary diagnoses were considered. To remove individuals with high probability of having nonidiopathic conditions, we excluded individuals with congenital malformations of genitalia (*ICD-10* codes Q50-Q56), and chromosomal abnormalities (*ICD-10* codes Q90-Q99) and censored follow-up from individuals receiving a cancer or carcinoma in situ diagnosis (C00-C99 and D00-D09) owing to the possibility of radiation therapy, a diagnosis of hypogonadism after ovarian treatment (E89.4), or a major surgical procedure on the genitalia. To capture more transient adverse events and primary ovarian failure not diagnosed as such, we also included a secondary outcome in the form of the composite outcome of the following diagnoses: N91.0-N91.2 (ie, amenorrhea) and N91.3-N91.5 (ie, oligomenorrhea). For this outcome, we also included referral diagnoses from primary health care.

### Health Care Use

Increased health care use may be associated with increased probability of being diagnosed with primary ovarian insufficiency owing to more opportunities for diagnostic workup or a lower threshold for seeking health care in general. To characterize health care use, we used all hospital contacts in the 5 years prior to study entry for all individuals in the study classified according to the number of contacts (ie, 0,1, or ≥2) with diagnoses in each of the 21 main chapters of the *ICD-10* classification. We then estimated propensity scores for each individual (ie, probability of vaccination during the study period) in a logistic regression model with health care use characteristics at study entry as dependent variables.^12^ The resulting propensities were then categorized by decile.

### Statistical Analysis

We followed individuals in the study cohort retrospectively from age 11 years or January 1, 2007, whichever event came later until the individual’s 35th birthday; January 1, 2017; emigration; death; or disappearance from the Civil Registration System, whichever event came first. HPV vaccination was considered a time-dependent variable with the possibility of individuals contributing follow-up as unvaccinated and vaccinated individuals. We considered individuals vaccinated after receipt of the first dose. Receiving HPV vaccines other than the 4HPV vaccine was considered a censoring event. Diagnoses of cancer or carcinoma in situ, hypogonadism after ovarian treatment, or major surgical procedures on the genitalia also resulted in censoring.

Outcomes (primary ovarian insufficiency or the composite outcome of amenorrhea and oligomenorrhea) and person-time of follow-up were aggregated by 4HPV vaccination status, age, calendar period, and propensity score decile. We then used Cox proportional hazards regression with age as the underlying time-scale to estimate HRs with 95% CIs comparing vaccinated and unvaccinated individuals. We estimated crude HRs (taking only age as the underlying time-scale into account) and adjusted HRs, which were estimated in models with the baseline hazard stratified by calendar period (in 1-year categories) and propensity score (in 10 categories). In addition to the main vaccinated and unvaccinated comparison, we conducted analyses subdividing vaccinated follow-up by time since last vaccination (ie, 0-90 days, 91 days-1 year, and ≥1 year), age at first vaccination (ie, ages 11-19 years and 20-34 years), and calendar period at first vaccination (ie, 2007-2011 and 2012-2016). Additionally, we conducted a stratified analysis comparing vaccinated and unvaccinated individuals by strata of propensity score (ie, 1st-3rd decile, 4th-7th decile, and 8th-10th decile).

We conducted 3 sensitivity analyses. (1) We re-estimated the association between 4HPV vaccination and primary ovarian insufficiency without exclusions and censoring owing to congenital malformations of genitalia, chromosomal abnormalities, a cancer or carcinoma in situ diagnosis, a diagnosis of hypogonadism after ovarian treatment, or a major surgical procedure on the genitalia. (2) We re-estimated the association between 4HPV vaccination and primary ovarian insufficiency using the earliest diagnosis of primary ovarian insufficiency or the composite outcome of amenorrhea and oligomenorrhea for individuals with both diagnoses. (3) We conducted a sensitivity analysis in which we started follow-up at age 15 years instead of age 11 years because we did not have information on age at menarche.

Data management and statistical analyses were conducted using R statistical software version 4.0.2 (R Project for Statistical Computing). We used the Epi package to construct follow-up intervals from the study cohort and the survival package for Cox regression. See the eAppendix in the Supplement for R code used to estimate the adjusted vaccination association with adjusted HR. *P* values were 2-sided, and the level of significance was set a *P* = .05. Data were analyzed from October 2020 to January 2021.

## Results

**Figure 1.**
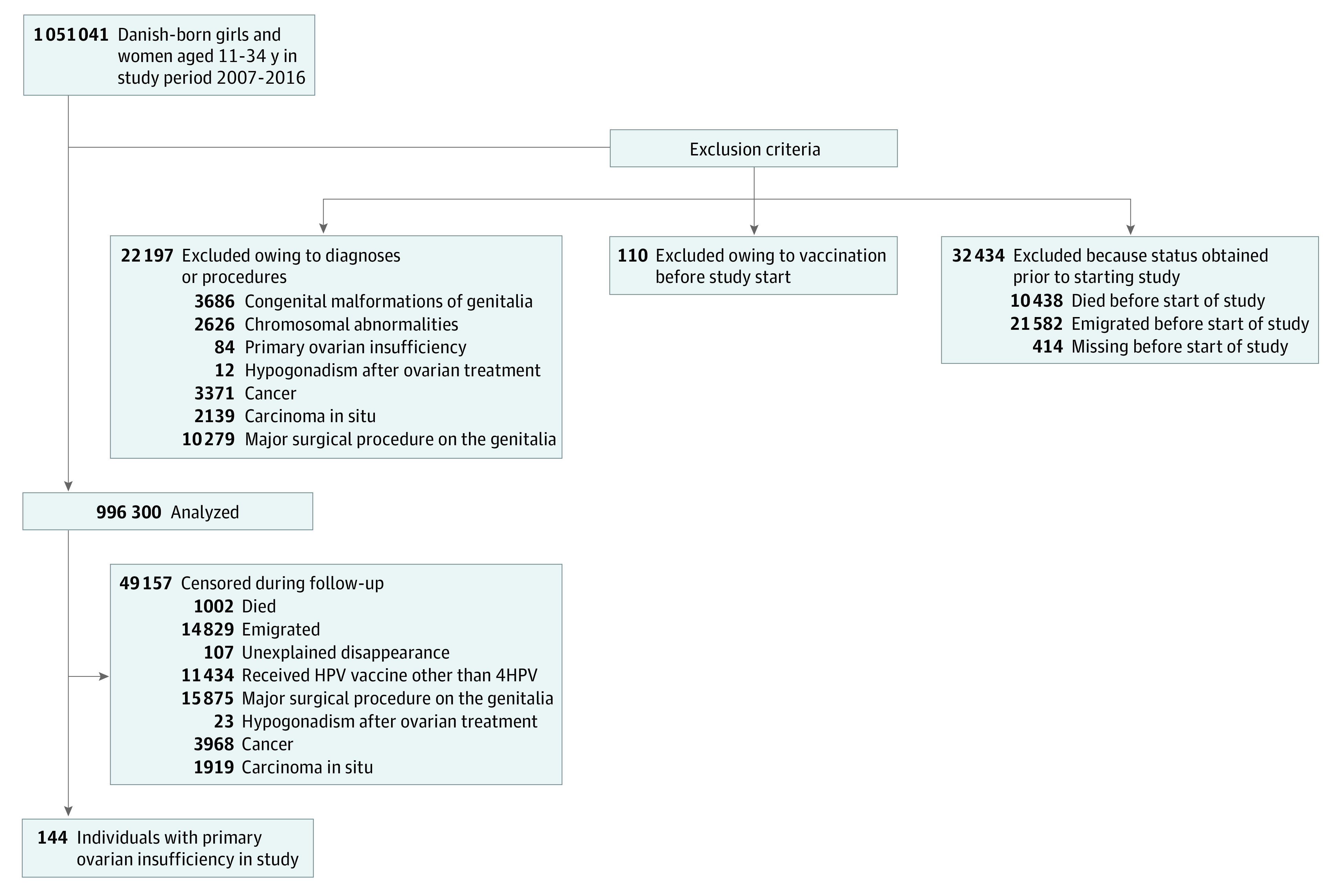
Study Flowchart

We identified 1 051 041 Danish-born girls and women aged 11 to 34 years during 2007 to 2016. We excluded 54 741 individuals before study start (Figure 1). The resulting study cohort of 996 300 individuals were followed for 6 781 166 person-years, and 144 diagnoses of primary ovarian insufficiency were ascertained, including among 54 vaccinated individuals and 90 unvaccinated individuals. During follow-up, 49 157 individuals were censored (Figure 1).

**Figure 2.**
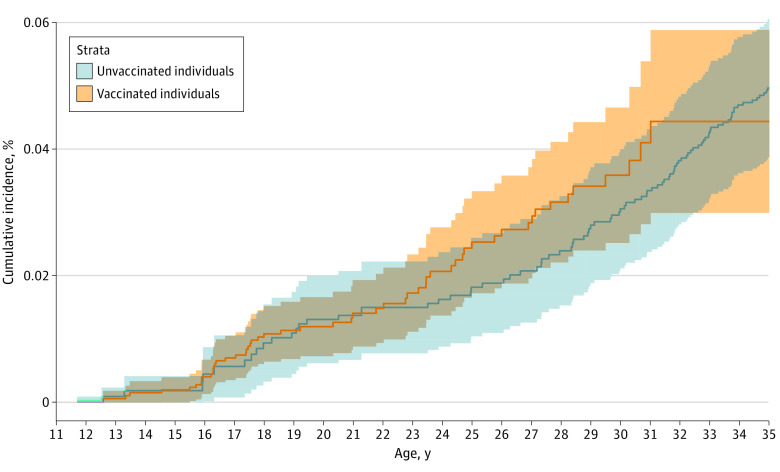
Cumulative Incidence of Primary Ovarian Insufficiency by Vaccination Status Shaded areas indicate 95% confidence bands.

The median (interquartile range [IQR]) age of primary ovarian insufficiency diagnosis was 26.94 (12.68) years. The cumulative incidence of primary ovarian insufficiency at age 19 years was 0.011% and 0.011% among vaccinated and unvaccinated individuals in the study, respectively (Figure 2). At age 34 years, the cumulative incidence was 0.044% and 0.047% among vaccinated and unvaccinated individuals in the study, respectively (Figure 2). Uptake of at least 1 dose of 4HPV vaccine in the study cohort occurred among 505 829 individuals (50.8%), with a median (IQR) age at first vaccination of 14.37 (10.02) years; 490 471 individuals were unvaccinated (49.2%). The birth cohort contributing the greatest proportion of vaccinated individuals in the study was the 1992 to 1996 group (147 396 individuals [29.1%]), while the birth cohort contributing the smallest proportion of vaccinated individuals was the 1972 to 1976 group (669 individuals [0.1%]) (Table 1). The age group with the highest number of individuals vaccinated was age 11 to 12 years (193 901 individuals [38.3%]). The calendar period with the highest uptake was 2011 to 2012 (170 327 individuals [33.7%]). The degree of health care use at study start was similar for vaccinated and unvaccinated individuals. The propensity score taking health care use into account had a *C* statistic of 0.70 for predicting treatment status in the study.^13^ The distributions of propensity scores by vaccination status were partially overlapping (eFigure 1 in the Supplement).

**Table 1.** Characteristics Among 996 300 Individuals in Study Cohort

Characteristic	Unvaccinated individuals, No. (%) (n = 490 471)	Vaccinated individuals, No. (%) (n = 505 829)
Birth cohort		
1972-1976	153 733 (31.3)	669 (0.1)
1977-1981	124 142 (25.3)	8768 (1.7)
1982-1986	73 711 (15.0)	49 711 (9.8)
1987-1991	27 015 (5.5)	116 916 (23.1)
1992-1996	15 571 (3.2)	147 396 (29.1)
1997-2001	11 147 (2.3)	145 738 (28.8)
2002-2005	85 152 (17.4)	36 631 (7.2)
Age at first vaccination, y		
11-12	NA	193 901 (38.3)
13-15	NA	111 192 (22.0)
16-19	NA	28 412 (5.6)
20-27	NA	156 736 (31.0)
28-34	NA	15 588 (3.1)
Calendar period at first vaccination		
2007-2008	NA	96 460 (19.1)
2009-2010	NA	117 662 (23.3)
2011-2012	NA	170 327 (33.7)
2013-2014	NA	101 511 (20.1)
2015-2016	NA	19 869 (3.9)
Health-care use, No. contacts^a^		
0	114 950 (23.4)	181 188 (35.8)
1	61 127 (12.5)	95 199 (18.8)
≥2	314 394 (64.1)	229 442 (45.4)

Abbreviation: NA, not applicable.

^a^ Number of hospital contacts in the 5 years before study entry.

In the main analysis of primary ovarian insufficiency, comparing vaccinated with unvaccinated individuals yielded an adjusted HR of 0.96 (95% CI, 0.55-1.68) (Table 2). The HR of vaccination did not differ significantly in periods after the last vaccination or by age or calendar period at first vaccination. In deciles of the propensity score, the adjusted HR was 0.58 (95% CI, 0.19-1.73) for the 1st through 3rd decile, 0.79 (95% CI, 0.35-1.80) for the 4th through 7th decile, and 1.98 (95% CI, 0.71-5.56) for the 8th through 10th decile. We evaluated the proportional hazards assumption using Schoenfeld residuals; residuals from adjusted models in Table 2 were consistent with the proportional hazards assumption being fulfilled for the vaccination outcomes (eFigure 2 in the Supplement).

**Table 2.** Risk of Primary Ovarian Insufficiency by Vaccination Status

Characteristic	Diagnoses, No.	Person-years	HR (95% CI)	
			Crude^a^	Adjusted^b^
Vaccination status				
Unvaccinated	90	4 021 340	1 [Reference]	1 [Reference]
Vaccinated^c^	54	2 759 826	1.16 (0.78-1.73)	0.96 (0.55-1.68)
Time since last vaccination, d				
0-90	6	304 262	1.64 (0.69-3.92)	1.01 (0.37-2.70)
91-365	11	449 138	1.76 (0.89-3.50)	0.99 (0.44-2.22)
≥366	37	2 006 425	1.01 (0.65-1.57)	0.94 (0.49-1.80)
Age at first vaccination, y				
11-19	32	2 024 850	0.98 (0.56-1.72)	0.77 (0.37-1.62)
20-4	22	734 976	1.34 (0.80-2.22)	1.15 (0.58-2.28)
Calendar period at first vaccination				
2007-2011	32	1 847 162	1.01 (0.61-1.66)	0.75 (0.39-1.45)
2012-2016	22	912 663	1.36 (0.82-2.24)	1.42 (0.67-2.99)

Abbreviation: HR, hazard ratio.

^a^ The underlying time scale is age.

^b^ The baseline hazard is stratified by calendar period in 1-year categories and propensity score in 10 categories by deciles.

^c^ Unaccinated individuals serve as reference group for all HRs.

In the analysis of the secondary composite outcome of amenorrhea and oligomenorrhea, comparing vaccinated with unvaccinated individuals yielded an adjusted HR of 1.09 (95% CI, 0.97-1.22) (Table 3). The HR of vaccination did not differ significantly in periods after the last vaccination or by age or calendar period at first vaccination. In deciles of the propensity score, the adjusted HR was 1.04 (95% CI, 0.83-1.30) for the 1st through 3rd decile, 1.14 (95% CI, 0.95-1.37) for the 4th through 7th decile, and 1.07 (95% CI, 0.88-1.29) for the 8th through 10th decile.

**Table 3.** Risk of Amenorrhea and Oligomenorrhea by Vaccination Status

	Diagnoses, No.	Person-years	HR (95% CI)	
			Crude^a^	Adjusted^b^
Vaccination status				
Unvaccinated^c^	1709	4 005 624	1 [Reference]	1 [Reference]
Vaccinated	1658	2 751 680	1.30 (1.21-1.40)	1.09 (0.97-1.22)
Time since last vaccination, d				
0-90	106	303 753	1.19 (0.97-1.45)	1.15 (0.92-1.43)
91-365	174	448 302	1.18 (1.01-1.39)	1.05 (0.87-1.27)
≥366	1378	1 999 625	1.33 (1.23-1.44)	1.09 (0.96-1.23)
Age at first vaccination, y				
11-19	1124	2 020 994	1.32 (1.20-1.45)	1.09 (0.94-1.26)
20-34	534	730 686	1.28 (1.15-1.42)	1.08 (0.94-1.25)
Calendar period at first vaccination				
2007-2011	1122	1 842 454	1.26 (1.16-1.38)	1.07 (0.94-1.21)
2012-2016	536	909 227	1.36 (1.23-1.51)	1.13 (0.97-1.32)

Abbreviation: HR, hazard ratio.

^a^ The underlying time-scale is age.

^b^ The baseline hazard is stratified by calendar period in 1-year categories and propensity score in 10 categories by decile.

^c^ Unaccinated individuals serve as reference group for all HRs.

In sensitivity analyses, we re-estimated the HR of primary ovarian insufficiency after 4HPV vaccination without exclusions and censoring owing to congenital malformations of genitalia, chromosomal abnormalities, a cancer or carcinoma in situ diagnosis, a diagnosis of hypogonadism after ovarian treatment, or a major surgical procedure on the genitalia. This yielded 6 970 880 person-years of follow-up and 193 diagnoses of primary ovarian insufficiency with an adjusted HR of 0.79 (95% CI, 0.49-1.28). There were 45 individuals with a diagnosis of primary ovarian insufficiency and preceding diagnoses of amenorrhea or oligomenorrhea. We re-estimated the HR of primary ovarian insufficiency after 4HPV vaccination using the earliest diagnosis of primary ovarian insufficiency or amenorrhea or oligomenorrhea. This yielded an adjusted HR of 1.32 (95% CI, 0.73-2.38). Because we had no information on age at menarche, we conducted a sensitivity analysis in which we started follow-up at age 15 years instead of age 11 years (and allowed individuals to be vaccinated at study start); this yielded an adjusted HR of 1.03 (95% CI, 0.58-1.83).

## Discussion

In this nationwide cohort study of 996 300 Danish girls and women, we found no association between 4HPV vaccination and primary ovarian insufficiency. The rate of primary ovarian insufficiency was not statistically significantly increased among vaccinated individuals compared with unvaccinated individuals overall or in time periods after vaccination; the rate of a composite outcome of oligomenorrhea and amenorrhea was not statistically significantly increased in the population either.

Few studies exist on the association between HPV vaccination and primary ovarian insufficiency.^14^ The proposed association originated from a case report on a 16-year-old Australian girl. Menarche occurred at age 13 years but was later followed by 17 months of oligomenorrhea and amenorrhea. The girl reported that menstrual irregularities had started after HPV vaccination.^15^ This case report was followed by a 2 cases presented by the same authors of the original report and a case series of 3 girls, 2 of whom were sisters, all HPV vaccinated before primary ovarian insufficiency diagnoses were made.^3,4^ A number of mechanisms have been proposed to explain the association, including autoimmune processes in response to the aluminum adjuvant in the vaccine and claimed ovarian toxic effects associated with polysorbate 80, used as an excipient in the vaccine.^5,6^ However, while postvaccination autoimmunity is theoretically plausible, a specific mechanism for primary ovarian insufficiency has not been substantiated, and polysorbate 80 levels from vaccine exposure are miniscule compared with toxic levels.^16,17^

From passive surveillance systems, we know that reporting of primary ovarian insufficiency is rare. The best estimates from national adverse event reporting databases range from 0.07 to 0.28 reports per million 4HPV doses distributed.^8,18^ A disproportionality analysis utilizing the Vaccine Adverse Event Reporting System database reported safety signals associated with 4HPV and menstruation irregularities.^19^ However, while safety signals generated from passive surveillance contribute little to the assessment of causality, they may warrant further investigation in analytical studies. Although 4HPV was not associated with a composite outcome of oligomenorrhea and amenorrhea in our study, our results suggest a statistically insignificant increase in the rate of this outcome. Our findings suggest that the increase is transient and is not associated with primary ovarian insufficiency diagnoses.

To our knowledge, the only analytical study of the rate of primary ovarian insufficiency diagnoses among individuals with the 4HPV vaccine is a cohort study of 199 078 female patients from a US health maintenance organization database.^7^ One of the strengths of this study was that it included careful medical record review, such as estimating the date of symptom onset. The authors confirmed 46 diagnoses of idiopathic primary ovarian insufficiency. However, 28 individuals had symptom onset after the HPV vaccine was available, and 1 individual had symptom onset after HPV vaccination, yielding an HR of 0.30 (95% CI, 0.07-1.36). Additionally, a recent cross-sectional study of survey data evaluated HPV vaccination and the rate of self-reported infertility and found no association.^20^ Although this is a reassuring result, the highly heterogeneous nature of infertility and the cross-sectional design preclude this study from adding significantly to the evidence on HPV vaccination and the rate of primary ovarian insufficiency.

The main strength of our study is the use of a nationwide cohort of girls and women with independent ascertainment of exposure and outcome status. Our study is the largest to date of 4HPV vaccination and primary ovarian insufficiency diagnosis, with 144 individuals, including 54 vaccinated individuals. It is the first, to our knowledge, to evaluate a composite outcome of oligomenorrhea and amenorrhea in a cohort study.

The HPV vaccines are cancer-preventing vaccines,^21^ and, thus, the lackluster uptake in many countries is disheartening. Our study provides much-needed support for the ovarian safety of the 4HPV vaccine, which is of key importance for clinical and public health personnel when addressing parental concerns about fertility issues associated with vaccination. There is currently little evidence for an association between 4HPV vaccination and primary ovarian insufficiency. On the contrary, the vaccine protects against infections that may decrease fertility.^22^

### Limitations

Our study has a number of limitations. First, we did not have access to dates of symptom onset, and it is likely that there is significant delay between symptom onset and diagnosis. If the length of this delay is not associated with vaccination, we expect there to be a number of individuals with onset before vaccination but diagnosis after vaccination, and these individuals may be falsely included as data for an association between vaccination and diagnosis. This may have biased our ratio toward an increase in the rate of diagnosis among vaccinated individuals. If vaccination is associated with a differentially decreased delay compared with no vaccination, this may further bias our results toward an increase because unvaccinated individuals may be less likely to be diagnosed within the study period. Second, it is likely that the ascertainment of primary ovarian insufficiency is incomplete in our Danish population. Not all individuals with the condition will be properly diagnosed in the hospital setting during the study period, and if this is differential, it may introduce bias. If vaccinated individuals use health care to a greater extent than unvaccinated individuals, our ratio may be biased toward an increase. Third, we did not include any information on menarche, and some of the youngest individuals in the study may thus not have been at risk of developing the outcome during follow-up. However, under the assumption that HPV vaccination is not associated with age at menarche, this immortal time is unlikely to bias our results, and we observed no association in an analysis starting at age 15 years instead of 11 years. Fourth, we did not have any information on oral contraceptive use in our study. Oral contraceptive use may prolong time to diagnosis and, if associated with HPV vaccination, decrease rates of diagnosis among vaccinated individuals, potentially masking an increased rate.

## Conclusions

These findings suggest that a moderate to large increase in the rate of primary ovarian insufficiency associated with vaccination is unlikely. However, given the rarity of the outcome in our study, the presence of a still clinically relevant increase cannot be excluded.

## Notes

Supplement. **eAppendix.** R Code Used to Estimate Adjusted Vaccination Association and Associated Output **eFigure 1.** Propensity Score Distribution Among Vaccinated and Unvaccinated Individuals **eFigure 2.** Schoenfeld Residual Plots for Vaccination Outcomes
