# Supplementary material for: Association Between Human Papillomavirus Vaccination and Primary Ovarian Insufficiency in a Nationwide Cohort
Source: JAMA Netw Open. 2021 Aug 26;4(8):e2120391. doi: 10.1001/jamanetworkopen.2021.20391 (PMC8391101; doi:10.1001/jamanetworkopen.2021.20391)
Supplement: Supplement. — eAppendix. R Code Used to Estimate Adjusted Vaccination Association and Associated Output eFigure 1. Propensity Score Distribution Among Vaccinated and Unvaccinated Individuals eFigure 2. Schoenfeld Residual Plots for Vaccination Outcomes [file jamanetwopen-e2120391-s001.pdf]

Supplemental Online Content

Hviid A, Myrup Thiesson E. Association between human papillomavirus vaccination and primary ovarian insufficiency in a nationwide cohort. *JAMA Netw Open*. 2021;4(8):e2120391. doi:10.1001/jamanetworkopen.2021.20391

eAppendix. R Code Used to Estimate Adjusted Vaccination Association and Associated Output

eFigure 1. Propensity Score Distribution Among Vaccinated and Unvaccinated Individuals

eFigure 2. Schoenfeld Residual Plots for Vaccination Outcomes

This supplemental material has been provided by the authors to give readers additional information about their work.

© 2021 Hviid A et al. *JAMA Network Open.*

eAppendix. R Code Used to Estimate Adjusted Vaccination Association and Associated Output

In the following, we provide the R-code and the accompanying output of the Cox proportional hazards model for the study's main estimate. This estimate is the hazard ratio comparing the hazards of primary ovarian insufficiency between human papillomavirus vaccinated and unvaccinated females, stratified by year in one-year intervals and propensity score in deciles, with age as the underlying time scale. When stratifying, the Cox proportional hazards model is fitted within each stratum, where separate baseline hazards are estimated for each level of the covariate.

```
main_model <- coxph(Surv(age, age + lex.dur, lex.Xst) ~ HPV_VACC_IND +
           strata(PROP_SCORE_IND) + strata(YEAR_IND), id = lex.id,
           data = POI_data)
summary(main_model)
```

```
Call:
coxph(formula = Surv(age, age + lex.dur, lex.Xst) ~ HPV_VACC_IND +
    strata(PROP_SCORE_IND) + strata(YEAR_IND), data = POI_data,
    id = lex.id)

  n= 11130303, number of events= 144

                 coef exp(coef) se(coef)      z Pr(>|z|)
HPV_VACC_IND1 -0.03664   0.96402  0.28439 -0.129    0.897

              exp(coef) exp(-coef) lower .95 upper .95
HPV_VACC_IND1     0.964      1.037    0.5521     1.683

Concordance= 0.493  (se = 0.01 )
Likelihood ratio test= 0.02  on 1 df,   p=0.9
Wald test            = 0.02  on 1 df,   p=0.9
Score (logrank) test = 0.02  on 1 df,   p=0.9
```

© 2021 Hviid A et al. *JAMA Network Open.*

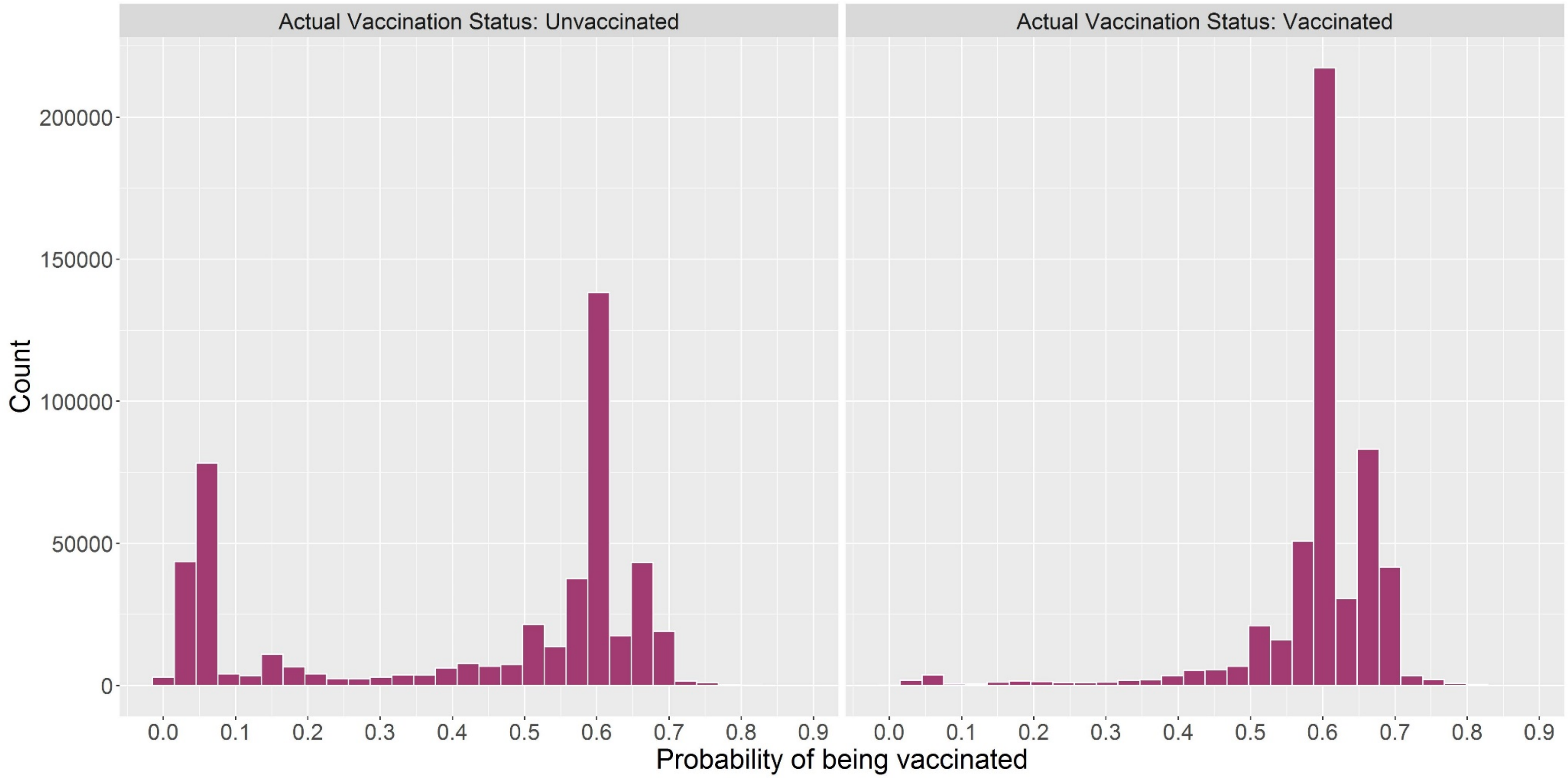

eFigure 1. Propensity Score Distribution Among Vaccinated and Unvaccinated Individuals

© 2021 Hviid A et al. *JAMA Network Open.*

eFigure 2. Schoenfeld Residual Plots for Vaccination Outcomes

The following plots show the Schoenfeld residuals for the adjusted models in Table 2 with age as the underlying time-scale. These residuals represent the difference between the observed covariate and the expected, given the risk set at that specific time. Visually, these should be flat and centered at zero. At the top of each plot is the Schoenfeld test for the correlation between the residuals and survival time, where zero correlation indicates that the model meets the proportional hazards assumption. A significant p-value indicates that the proportional hazards assumption is not satisfied.

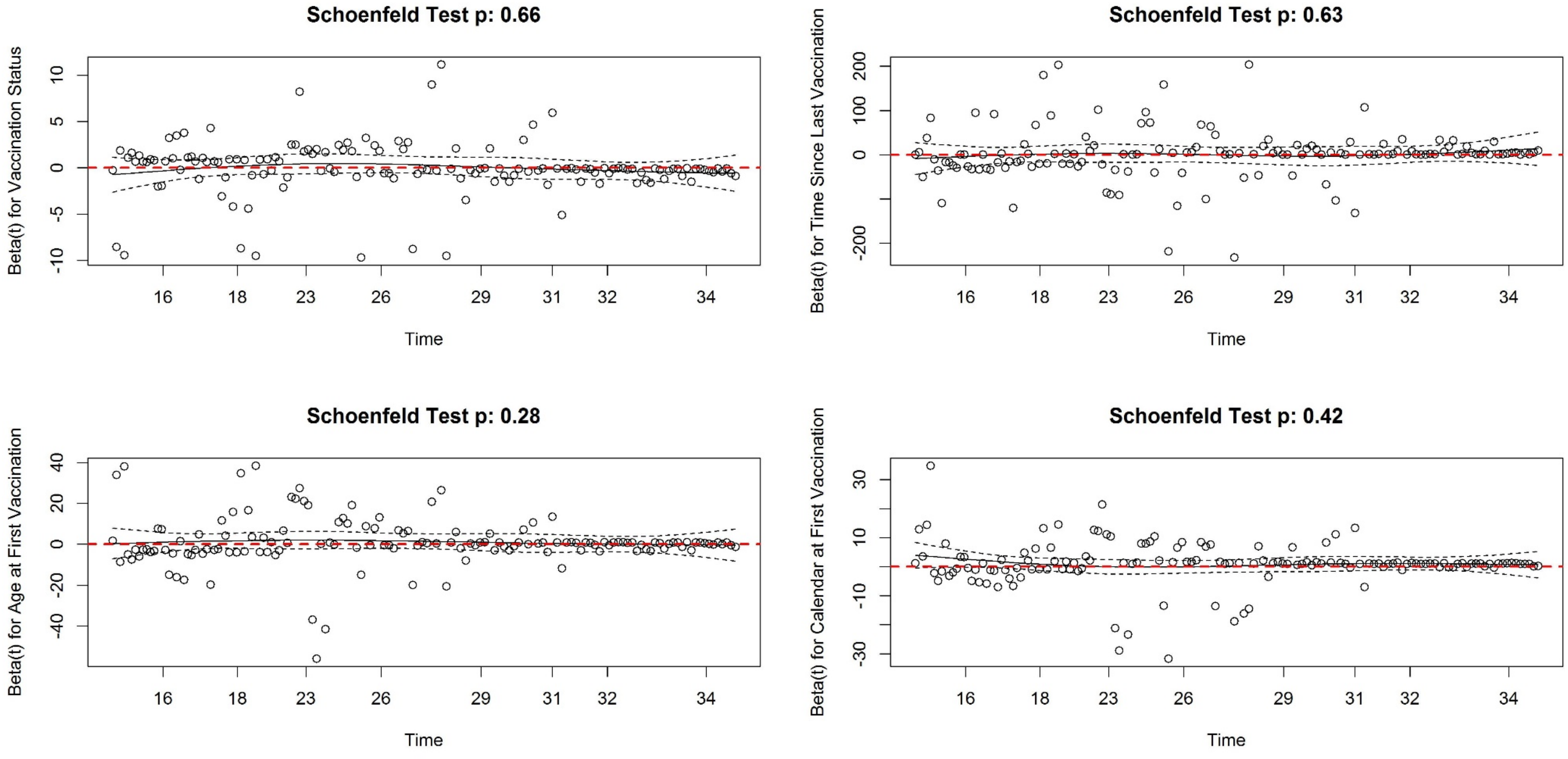

© 2021 Hviid A et al. *JAMA Network Open.*
